# Bacterial Aetiology and Antibiotic Susceptibility Profile of Post-Operative Sepsis among Surgical Patients in a Tertiary Hospital in Rural Eastern Uganda

**DOI:** 10.9734/MRJI/2018/41690

**Published:** 2018-06-20

**Authors:** Masifa George, Jacob Stanley Iramiot, Rita Muhindo, Peter Olupot-Olupot, Ann Nanteza

**Affiliations:** 1 School of Bio-security, Biotechnical & Laboratory Sciences, College of Veterinary Medicine, Animal Resources & Bio-security, Makerere University, Kampala, Uganda.; 2 Mbale Clinical Research Institute (MCRI), P.O.Box 1966, Mbale, Uganda.; 3 Faculty of Health Sciences, Busitema University, Mbale Campus, P.O.Box 1460, Mbale, Uganda.

**Keywords:** Antibiotic, antibiotic resistance, antibiotic susceptibility, post-operative sepsis

## Abstract

**Background:**

Post-operative wound sepsis remains a surgical challenge of public health concern constituting approximately 20% of the health care-associated nosocomial infections. This study aimed at determining the prevalence and antimicrobial resistance patterns of bacterial pathogens isolated from post-operative wound infections at Mbale Regional Referral Hospital.

**Materials and Methods:**

This was a descriptive cross-sectional study conducted from June to October 2015. Study participant samples were sub-cultured upon reception in the Microbiology laboratory and the isolated bacterial pathogens were analysed. Phenotypic antimicrobial susceptibility profiles were determined using the Kirby-Bauer method. Interpretation of the zone diameters was done following the Clinical and Laboratory Standards Institute guidelines. Phenotypic screening for Methicillin-resistant *Staphylococcus aureus* (MRSA) was performed using oxacillin (1 μg). D-test was also performed for phenotypic screening of inducible clindamycin resistant *Staphylococcus aureus*, Data were entered into Microsoft Excel and analysed using IBM SPSS statistics (version 16).

**Results:**

Overall post-operative sepsis was 69/80 (86.2%) with *Staphylococcus aureus* as the most predominant organism 41/104 (39.4%) followed by *Escherichia coli* 22/104 (21.2%) and *Klebsiella* species 15/104 (14.4%). Of the 41/104 isolated *Staphylococcus aureus*, 27/41(65.9%) were MRSA strains and 5/41 (12.2%) were inducible clindamycin resistant *Staphylococcus aureus* strains. The isolated *Staphylococcus aureus* was resistant to multiple drugs though susceptible to vancomycin and clindamycin. In addition, none of the isolated *Enterococci species* was vancomycin resistant. Although most of the isolated Gram-negative organisms were sensitive to imipenem, resistance was observed for tetracycline, trimethoprim/sulphamethoxazole, and ceftriaxone.

**Conclusion:**

*Staphylococcus aureus* was the most common causative agent associated with postoperative sepsis with most of the strains being MRSA. Multi-drug resistance was observed in 63/104 (60.6%) of the isolated organisms in our study. Hence the need to better develop and strengthen antimicrobial stewardship programs as well as to understand the carriage of antimicrobial resistance genes among these organisms.

## 1. INTRODUCTION

Post-operative wound infection is an infection that develops within 30 days after an operation or within one year if an implant was placed, and the infection appears to be related to the surgery [1]. Globally, post-operative wound sepsis is a growing public health concern constituting approximately 20% of all the health care-associated infections [2]. Developing countries are the most affected, possibly due to limited resources including equipment, personnel and infrastructure to handle the operative cases [3]. Whereas there are potentially a number of sources for post-operative wound infections, hospital-acquired sources or nosocomial infections remain the commonest, and their frequencies are dependent on the hospital setting [4,5].

Post-operative infections have been characterized as either superficial or deep cut infections depending on the type of the wound. The associated factors include nature of the infecting organism, host resistance, nature of the surgery, dose and antibiotic use, and anaesthesia methods [6]. In a number of infections, the causative pathogens implicated in these cases come from the endogenous flora of the patient’s skin and mucous membrane [7]. Some of the bacterial pathogens isolated in these infections include *Staphylococcus aureus*, *Enterobacteriaceae*, Coagulase Negative *Staphylococci* (CoNS), *Enterococci spp* and *Pseudomonas aeruginosa* [8,9].

Earlier prevalence studies have shown that *Staphylococcus aureus* and *Pseudomonas aeruginosa* were the most isolated organisms in post-operative wound infections accounting for 20–40% and 5–15% respectively [10], with the variation indicating geographical and temporal relationships [11]. Nosocomial infections and post-operative wound sepsis have remained a major cause of morbidity and death among the operated patients [12].

Post-operative wound infection control practices have been proposed, evaluated and recommended for instance, improved operating room ventilation, sterilization methods, use of barriers, surgical techniques, antiseptic measures, and use of antimicrobial prophylaxis [7]. However, in settings where studies have been done, data indicates that 30–50% of antibiotics are prescribed for post-operative wound prophylaxis, and 30–90% of the prophylaxis is given empirically which is inappropriate and in some instances prolonged [13]. The norms and inappropriate practices of empirical treatment have resulted in the emergence of pathogenic drug-resistant bacteria [13]. With recent advances to combat the alarming issue of antimicrobial resistance through antimicrobial stewardship programs, inadequate information still exists in the most affected regions of the world, especially the resource-limited settings. In this study, we determined the prevalence of common bacterial pathogens causing post-operative wound infections and their antibiotic culture and sensitivity patterns at Mbale Regional Referral Hospital in Eastern Uganda.

## 2. MATERIALS AND METHODS

This was a descriptive cross-sectional study conducted between June and October 2015 at Mbale Regional Referral Hospital in Eastern Uganda. The hospital provides services to both urban and rural catchment population in the 9 districts with approximately 4.5 million people in the region. The health services are free of charge.

### 2.1 Study Procedures

Informed consent was obtained from study participants and recruitment into the study was on the basis of the participants who met the inclusion criteria; post-operative patients with confirmed surgical site infection at the hospital’s general surgery and gynaecology wards during the study period. Study participants’ demographic data were collected using a pre-tested interviewer-administered the semi-structured questionnaire. Convenience sampling techniques were used for all the study participants with wound sepsis based on the evidence of post-operative wound infection during the study period. Pus swabs were collected aseptically before wounds were cleaned and dressed by the attending nurses. The samples were aseptically collected using sterile cotton swabs from infected wound sites and immediately transported to Mbale regional referral hospital microbiology laboratory for culture and drug susceptibility testing.

### 2.2 Laboratory Procedures

Upon reception, the pus swabs were subcultured on MacConkey, blood and chocolate agar for maximum recovery. Chocolate agar plates were placed in a candle jar and incubated with other plates at 37°C for 24 hours.

Plates were examined for growth aerobically to identify potential pathogens based on their characteristic morphological appearance on the respective media. Gram staining was done and pathogen identity was confirmed by a series of conventional biochemical tests that were available. Members from the family Enterobacteriacea and other Gram-negative rods were identified using in-house Triple Sugar Iron (TSI), oxidase, indole, citrate, chromogenic agar as recommended by other authors [14] whereas the Gram-positive bacteria were identified by bound coagulase, catalase and chromogenic agar [15].

Antibiotic susceptibility testing was performed using the Kirby- Bauer disc diffusion test on Muller Hinton agar [16]. Muller Hinton agar (MHA) plates were evenly inoculated with bacterial suspensions standardised to a 0.5 McFarland standard using a sterile cotton swab and the set up was incubated aerobically at 37°c for 24 hours. The following drugs were tested; imipenem (10 μg), trimethoprim/sulphamethoxazole (1.25/23.75 μg), ceftriaxone (30 μg), tetracycline (10 μg), chloramphenicol (30 μg), gentamicin (10 μg), vancomycin (30 μg), clindamycin (2 μg), erythromycin (15 μg), penicillin G (10 μg), and oxacillin (1 μg). The radius of the zone of inhibition was measured using an inhibition zone ruler and results were interpreted as resistant or sensitive in accordance to the Clinical Laboratory Standard Institute (CLSI) guidelines for standard performance of antimicrobial disc susceptibility tests [17].

Phenotypic screening for methicillin resistance was performed using oxacillin (1 μg) and an inhibition zone diameter of ≤10 mm was considered resistant for oxacillin [17]. Phenotypic screening for Inducible clindamycin resistant *Staphylococcus aureus* was also done using the D-test. The D- test was performed on all *Staphylococcus aureus* isolates by placing both clindamycin and erythromycin discs 15 mm apart from the centre of the MHA plate. Flattening on the side of erythromycin was read as inducible clindamycin resistance while a zone of clearance towards the side of erythromycin was read as clindamycin sensitive as described by Fiebelkorn et al. [18].

### 2.3 Statistical Analysis

Variables from the demographic and laboratory data were entered into excel, cleaned and exported to IBM SPSS statistics (version 16) for statistical analysis. Continuous variables were described as mean (± Standard Deviation). Categorical variables were analyzed using the Pearson Chi-square test and results presented in tables as proportions.

## 3. RESULTS

This study enrolled a total of 80 study participants with post-operative wound infections and corresponding pus swab specimens were studied. Of the 80 study participants, 61/80 (76.2%) were female and 19/80 (23.8%) were male. The study participants’ ages ranged between 2 to 80 years, with a mean age of 26.55 (*SD*= ±14.19) years. The infection rate was most prevalent in the age group of 19–35 years (51.2%), least prevalent in the age groups below 19 years (16.2%) and above 35 years (18.8%); more in females (66.2%) than in males. This was statistically significant with *p*=0.038 and *p*<0.05 respectively. Of the 80 non-repeat wound swabs collected, 69/80 (86.3%) were culture positive and a total of 104 bacterial pathogens were isolated with none being anaerobic. Single pathogenic bacterial isolates were recovered from 54/80 (67.5%) of the samples whereas 26/80 (32.5%) samples had poly-microbial infection. It was found that poly-microbial infections were significantly less common than the single pathogenic infections; *F* (2, 14.2) =16.93, *p*<0.05.

Table 1 illustrates the frequency of the bacterial pathogens isolated. Gram negative and Gram-positive bacteria recovered constituted 60/104 (57.8%) and 44/104 (42.2%) respectively of the bacterial isolates. *Staphylococcus aureus* was the predominantly isolated pathogen accounting for 41/104 (39.4%).

Tables 2 and 3 illustrate the frequency of antimicrobial susceptibility patterns of the isolated Gram-negative and Gram-positive bacterial pathogens during the study. Analysis of species specific resistance rates indicated that *Staphylococcus aureus* was resistant to penicillin G 33/41(80.5%), erythromycin 30/41(73.2%) and oxacillin 27/41(65. 9%) (Table 2). On the other hand, resistance to gentamicin 23/41(56.1%) and ceftriaxone 24/41 (58.5%) were observed (Table 2). Both *Staphylococcus aureus* and *Enterococcus spp* were susceptible to vancomycin and clindamycin. All Gram-negative bacteria were susceptible to imipemen, except 1/9 (11.1%) and 1/22 (4.5%) of the *Pseudomonas species* and *Escherichia coli* respectively. Most of the Gram negative bacteria isolated showed a multi-drug resistance pattern 44/60 (73.3%).

Of the 41 *Staphylococcus aureus* that were phenotypically screened for MRSA, 27/41 (65.9%) were positive for MRSA. The MRSA strains were susceptible to vancomycin and clindamycin as shown in (Table 2) above though 3/27 strains of MRSA were vancomycin resistant by the disc diffusion method.

## 4. DISCUSSION

Findings in this study showed that the isolation rate of 69/80 (86.2%) for the bacterial pathogens was higher compared to previous studies conducted in the same region [19,20]. However, Studies conducted elsewhere in Nepal and Nigeria reported similar high isolation rates of 80% and 86.13% respectively [21,22]. Information from our study showed that Gram-negative organisms were the most commonly isolated organisms in post-operative wound infections accounting for 60/104 (57.8%) which was higher compared to Gram-positive organisms which accounted for 44/104 (42.2%) as shown in (Table 1). Studies in Ethiopia and Iran showed a similar prevalence rate for Gram positive and Gram negative organisms [23,24]. However, reports from the developed countries like the US [7] documented that Gram positive bacteria were the most isolated pathogens. The source of origin remains unknown though exogenous sources from the Hospital environment could be potential niches.

In agreement with reports of previous studies, the most predominant bacterial pathogen was *Staphylococcus aureus* 41/104 (39.4%) and *Escherichia coli* 22/104 (21.2%); causing most of the post-operative wound infections as indicated in (Table 1) [2,9,25]. The endogenous nature of *Staphylococcus aureus* as a normal flora on the skin and becoming opportunistic in wound infections explains why it was associated with most of the surgical infections. The proportions and distribution patterns of the bacterial pathogens were similar to results documented by previous studies conducted in North-western Tanzania, Iran, and Kenya [9,26,27]. Information from the study is a reflection of lack of adequate post-operative care and failure to maintain theatre sterility during surgical procedures, inadequate infection control due to poor hygiene, resource and structural constraints.

Information from this study also provides insights into the problem of resistance in bacterial pathogens in Eastern Uganda. Findings in our study demonstrated that, in general, bacterial isolates associated with postoperative wound sepsis were resistant to most antibiotics that were commonly prescribed in the eastern region of Uganda (Tables 2 and 3). High rates of resistance to penicillin G 33/41 (80.5%) by *Staphylococcus aureus* were observed in the study. A similar study in Tanzania by Fehr et al. documented a high resistance rate of 95% to penicillin [28]. The proportion of MRSA 27/41 (65.9%) was high among the *Staphylococcus aureus* and the strains were susceptible to Vancomycin 38/41 (92.7%) with a noted vancomycin resistance among 3/27 (11.1%) MRSA strains. A similar study conducted in India by Kamat et al. [29] also reported a high MRSA isolation rate of 71%. The prescription of β-lactam antibiotics as first-line antibiotics may be rendered ineffective in future due to the high isolation rate of MRSA strains.

Enterobacteriaceae displayed a multidrug resistance pattern 44/60 (73.3%) to most of the antibiotics that were tested in the study (Table 5) though they were more sensitive to imipenem. This could have been due to mutation of the bacteria to adapt by developing resistance mechanisms against the commonly prescribed drugs. Imipenem is not a commonly used drug because it is expensive as well as the mode of administration of the drug that requires a qualified personnel. Gram-negative bacilli were variably resistant to the drugs tested as indicated in (Table 3) with most organisms being sensitive to imipemen and chloramphenicol. *Escherichia coli and Klebsiella* species showed resistance to gentamicin, ceftriaxone, tetracycline, and sulphamethoxazole/trimethoprim. This could be explained by the overuse and misuse of the antimicrobials in the region. In our study, resistance to ceftriaxone was noted among both Gram-negative and Gram-positive organisms as shown in both (Tables 2 and 3). This could have been attributed to the use of the drug as first-line broad-spectrum empirical treatment in the hospital in combination with other drugs.

## 5. CONCLUSION

Our study showed that most of the postoperative infections were caused by multi-drug resistant organisms 63/104 (60.6%) at Mbale Regional Referral Hospital hence posing a challenge to antibiotic therapy. MRSA strains were isolated in higher proportions than previously reported in Uganda. Therefore, there is need to strengthen infection control surveillance in the region to generate baseline information to better guide antimicrobial prescription. In addition, strengthening antimicrobial stewardship programs would be valuable in order to understand the carriage of antimicrobial resistance genes among these organisms.

## Figures and Tables

**Table 1.** Type and frequency of bacterial pathogens

Bacteria isolated	Frequency (n)	Prevalence (%)
*Staphylococcus aureus*	41	39.40
*Escherichia coli*	22	21.20
*Klebsiella* spp	15	14.40
*Pseudomonas* spp	9	8.70
*Proteus* spp	6	5.80
*Enterobacter* spp	6	5.80
*Citrobacter* spp	2	1.90
*Enterococcus* spp	3	2.80
Total	104	100.00

**Key**: n=number of strains isolated, %=percentage isolation

**Table 2.** Antibiotic resistance patterns for Gram positive bacterial isolate

Antibiotic tested	Staphylococcus aureus n(%)	*Enterococcus spp* n(%)
GM	23(56.1)	0(0.0)
CRO	24 (58.5)	0(0.0)
VA	3 (7.3)	0(0.0)
DA	9 (22.0)	0(0.0)
E	30 (73.2)	0(0.0)
Ox	27 (65.9)	
P	33 (80.5)	1 (33.3)

**Key:** GM=gentamicin, CRO=ceftriaxone, VA=vancomycin, DA=clindamycin, E=erythromycin, OX=oxacillin, P=penicillin G, n=number of resistant strains, %=percentage

**Table 3.** Antibiotic resistance patterns for gram negative bacterial isolates

Antibiotic tested	*E. coli* n(%)	*Klebsiella* spp n(%)	*Pseudomonas* spp n(%)	*Proteus* spp n(%)	*Enterobacter* spp n(%)	*Citrobacter* spp n(%)
GM	15(68.9)	13(86.7%)	7(77.8%)	5(83.3%)	5(83.3%)	0(0.0)
CRO	18(81.8%)	13(86.7%)	7(77.8%)	4(66.7%)	4(66.7%)	0(0.0)
C	6(27.3%)	13(86.7%)	4(44.4%)	3(50.0%)	4(66.7%)	1(50.0%)
IPM	1(4.5%)	0(0.0)	1(11.1%)	0(0.0)	0(0.0)	0(0.0)
TE	18(81.8%)	12(80.0%)	7(77.8%)	4(66.7%)	2(33.3%)	0(0.0)
S XT	19(86.4%)	14(93.3%)	7(77.8%)	100.0%	4(66.7%)	1(50.0%)

**Key:** GM= gentamicin, CRO= ceftriaxone, C= chloramphenicol, IPM= imipenem, TE= tetracycline, SXT= trimethoprim/sulphamethoxazole, n=number of resistant isolates, %=percentage

**Table 4.** Multi-drug resistance among the isolated Gram positive bacterial isolates

Isolates	Non-MDR n(%)	R3 n(%)	R4 n(%)	R5 n(%)	R6 n(%)	R7 n(%)
*Staphylococcus aureus*	15(36.6%)	6(14.6%)	12(29.3%)	6(14.6%)	1 (2.4%)	1 (2.4%)
*Enterococcus species*	3(100.0%)	0 (0.0%)	0 (0.0%)	0 (0.0%)	0 (0.0%)	0 (0.0%)

**Key:** R3= resistant to 3drug classes, R4= resistant to 4drug classes, R5= resistant to 5drug classes, R6= resistant to 6drug classes, R7= resistant to 7drug classes

**Table 5.** Multi-drug resistance among the isolated Gram negative bacterial isolates

Isolates	NOn-MDR n(%)	R3 n(%)	R4 n(%)	R5 n(%)	Total n(%)
*Klebsiella species*	1 (7.7%)	1 (11.1%)	4 (30.8%)	9 (36.0%)	15(14.4%)
*Escherichia coli*	5 (38.5%)	6 (66.7%)	4 (30.8%)	7 (28.0%)	22(21.2%)
*Pseudomonas species*	2(15.4%)	0 (0.0%)	3(23.1%)	4(16.0%)	9 (8.7%)
*Proteus species*	1 (7.7%)	2 (22.2%)	0 (0.0%)	3(12.0%)	6 (5.8%)
*Enterobacter species*	2(15.4%)	0 (0.0%)	2(15.4%)	2 (8.0%)	6 (5.8%)
*Citrobacter species*	2(15.4%)	0 (0.0%)	0 (0.0%)	0 (0.0%)	2(1.9%)

**Key:** R3= resistant to 3 drug classes, R4= resistant to 4 drug classes, R5= resistant to 5 drug classes
